# Prevalence and influencing factors of pruritus in maintenance hemodialysis patients in China: a meta-analysis

**DOI:** 10.1186/s12882-025-04163-7

**Published:** 2025-05-29

**Authors:** Mengjiao Li, Ping Jiang, Xujie Zhao, Yuping Ning, Liwen Huang

**Affiliations:** 1 Shanghai University of Traditional Chinese Medicine, Shanghai, 201203 China; 2 Shanghai Pudong New District People’s Hospital, Shanghai, 200120 China; 3 Shihezi University, Shihezi City, Xinjiang Uygur Autonomous Region 832000 China

**Keywords:** Hemodialysis, Pruritus, Prevalence, Influencing factors, Chinese, Meta-analysis

## Abstract

**Objective:**

To explore the prevalence and influencing factors of Chronic Kidney Disease-associated Pruritus (CKD-aP) among maintenance hemodialysis (MHD) patients in China through a meta-analysis.

**Methods:**

A systematic computerized search was conducted across ten databases, including CNKI, VIP, Wanfang, PubMed, Web of Science, and The Cochrane Library, to identify studies on the prevalence and influencing factors of pruritus among Chinese hemodialysis patients up to January 2025. Two independent graduate students conducted literature screening, data extraction, and bias risk assessment for the included studies. Data analysis was performed using Stata 17.0 software.

**Results:**

A comprehensive meta-analysis of 27 studies involving 10,346 participants identified 5,968 cases of CKD-ap among hemodialysis patients, examining 30 potential influencing factors. The meta-analysis revealed that the prevalence of CKD-ap in China was 52%, with 26% of patients experiencing mild CKD-ap, 22% moderate CKD-ap, and 8% severe CKD-ap. Factors associated with an increased risk of CKD-ap included duration of dialysis treatment (OR = 1.51, 95% CI: 1.15–1.88), primary disease (OR = 1.43, 95% CI: 0.87–1.99), dry skin disease (OR = 2.46, 95% CI: 1.74–3.19), phosphorus (OR = 1.18, 95% CI: 0.55–1.81), Ca - P product (OR = 2.18, 95% CI: 1.14–3.22), C - reactive protein (CRP) (OR = 1.14, 95% CI: 0.78–1.51), iPTH (OR = 2.45, 95% CI: 0.81–4.09), β_2_ - MG(OR = 2.24, 95% CI: 0.94–3.53), and SCr (OR = 1, 95% CI: 1.001–1.005). Conversely, factors including blood calcium levels (OR = 0.33, 95% CI: 0.31–0.35), dialysis modality (OR = 0.54, 95% CI: 0.23–0.85), and Kt/V (OR = 0.60, 95% CI: 0.24–0.96) were associated with a reduced risk of CKD-ap.

**Conclusion:**

This meta-analysis demonstrates a high prevalence of CKD-ap among Chinese MHD patients, underscoring the urgent need for improved awareness, preventive interventions, and management strategies in clinical practice.

**Clinical trial number:**

Not applicable.

**Trial number:**

The study was registered in PROSPERO: No. CRD42024562865.

**Supplementary Information:**

The online version contains supplementary material available at 10.1186/s12882-025-04163-7.

In recent years, the incidence of end-stage kidney disease (ESKD) in China has increased significantly, with approximately 2% of chronic kidney disease (CKD) patients progressing to ESKD annually [1]. However, ESKD represents the terminal stage of CKD or acute kidney injury (AKI), characterized by a severe and rapid decline in kidney function. The global prevalence of ESKD is estimated to be around 0.07%. In China, approximately 132 million individuals are diagnosed with CKD, with about 2% progressing to ESKD annually [1–2]. Without renal replacement therapy (RRT), ESKD patients are at high risk of developing life-threatening complications. Currently, approximately 2 million patients with ESKD worldwide are treated with hemodialysis for survival [3], whereas approximately 850,800 patients in China require long-term hemodialysis [4]. ESKD typically has an insidious onset, and over time, patients often develop extensive physical and psychological complications, accompanied by significant stress.

Chronic kidney disease-associated pruritus (CKD-aP), also known as uremic pruritus (UP), is a common, distressing, and often overlooked complication in patients with CKD or ESRD [5–6]. Most uremic patients, having lost renal metabolic function, require maintenance hemodialysis (MHD) to sustain life. While MHD improves survival rates in ESRD patients, it is often complicated by pruritus-related issues, such as xerosis (dry skin), and immune dysfunction, which can exacerbate symptoms and contribute to skin infections [7]. The pathogenesis of CKD-ap, a stress-induced skin or mucosal response, is multifactorial [8–9] and may involve the accumulation of uremic toxins, inflammatory responses, opioid receptor activation, and skin desiccation [10–11]. It is accompanied by numerous adverse effects, such as negative emotions, fatigue, and sleep disorders, severely affecting the life quality of patients [12–13]. Patients may also scratch their skin, resulting in wounds and infections [14–15], which can further increase the risk of mortality.Medical advancements have notably enhanced the quality of life for MHD patients. However, the incidence of CKD-ap has gradually increased owing to inadequate awareness among medical professionals regarding CKD-ap in MHD patients [10]. Further research is needed to clarify the pathogenetic mechanisms of CKD-ap.

Over the past decade, cohort studies by international researchers have reported an incidence of CKD-ap ranging from 40 to 90% in MHD patients and from 19 to 29% in non-MHD patients. However, clinical workers often overlook the adverse effects and burdens caused by CKD-ap in MHD patients [16–17]. Recent foreign studies have indicated that the incidence of moderate to severe CKD-ap is approximately 40% [18]. In recent years, more attention has been paid to CKD-ap, with European countries such as Spain and France developing interventions and treatment methods to prevent or reduce its occurrence [19–20]. Chinese studies on MHD patients have revealed that approximately 78% of MHD patients have CKD-ap, and 49.22% of them suffer from moderate to severe CKD-ap [21]. According to some scholars [22–23], the incidence of CKD-ap in Chinese MHD patients ranges from 20 to 98%, which is significantly higher compared to that reported in other studies [18]. This discrepancy may be attributed to the insufficient attention to CKD-ap in MHD patients in China, coupled with a lack of clinical trials and interventions. Consequently, treatment often only commences after symptoms appear, highlighting a lack of awareness regarding CKD-ap prevention. Therefore, clarifying the incidence of CKD-ap in Chinese patients with MHD and its associated factors is crucial for improving skin management and the quality of life of t this patient population. Currently, numerous related studies exist; however, they show significant discrepancies in results due to differences in sample size, region, and assessment tools. To this end, the present meta-analysis was conducted to systematically evaluate CKD-ap prevalence and its associated factors in Chinese MHD patients, aiming to inform future preventive strategies.

## Data and methods

### Data source

#### Literature retrieval

Electronic databases including CNKI, VIP, SinoMed, Wanfang, PubMed, Web of Science, Embase, The Cochrane Library, Ovid, and Scopus were searched for papers on UP in Chinese MHD patients from their inception to January 2025. The literature search was conducted using a combination of controlled vocabulary (subject terms) and free-text keywords. Subject terms included “renal dialysis*, hemodialysis*, dialysis, kidney, renal dialysis, hemodialysis, maintenance hemodialysis, extracorporeal dialysis, MHD”; “itch*, itchiness, pruritus”; “factors, influencing factors, associated factors, correlates, risk factors, predictors, causes”; and “Chinese OR China OR Sinkiang OR Inner Mongolia OR Hong Kong OR Taiwan OR Macao.” The PubMed search strategy was employed for reference (Table 1).

**Table 1:** Search strategies of pubmed databases

#1	Renal Dialysis[Mesh] OR (hemodialysis[Title/Abstract] OR dialysis[Title/Abstract] OR extracorporeal dialysis[Title/Abstract] OR renal dialysis[Title/Abstract] OR blood dialysis[Title/Abstract] OR maintenance hemodialysis[Title/Abstract] OR MHD[Title/Abstract]))
#2	Pruritus[Mesh] OR (Pruritis*[Title/Abstract]OR Itching*[Title/Abstract]OR Itch[Title/Abstract])
#3	risk factor[Mesh] OR (factor[Title/Abstract] OR influence factors[Title/Abstract] OR associated factors[Title/Abstract] OR relevant factors[Title/Abstract] OR risk factors[Title/Abstract] OR predicted factor[Title/Abstract] OR reason[Title/Abstract]))
#4	Chinese OR China OR Sinkiang OR Inner Mongolia OR HongKong OR TaiWan OR MaCao
#5	#1 AND #2 AND #3 AND #4

### Inclusion and exclusion criteria

#### Inclusion criteria

(1) Research type: Cross-sectional and longitudinal studies. (2) Research target: Chinese MHD patients. (3) Assessment tool: Meeting the diagnostic criteria for Chronic Kidney Disease-associated Pruritus (CKD-aP). (4) Outcome indicators: Incidence of CKD-ap in Chinese MHD patients and its associated factors; odds ratios (OR) and 95% confidence intervals (CI) reported in the literature. (5) Languages of literature: Chinese and English.

#### Exclusion criteria

(1) Studies with duplicate data. (2) Case reports, reviews, conference papers, and comments. (3) Literature with abnormal or incomplete data. (4) Research with unreasonable design or poor quality. (5) Repeated publications and studies whose full text could not be acquired.

### Literature selection and data extraction

*Literature Selection* Two researchers independently conducted a three-stage literature selection process, beginning with title screening, followed by abstract evaluation, and finally full-text review to determine eligibility. Any disagreements or discrepancies were resolved through consultation with a third researcher.

*Data Extraction* Two researchers independently extracted data from the literature, including authorship, publication year, research site, duration of the study, study design, patient gender, duration of dialysis, sample size, number of patients, prevalence, and associated factors. Data extraction was performed and summarized using Microsoft Excel, with cross-verification by the two researchers. Any discrepancies were resolved through consultation with a third researcher.

### Quality evaluation

The quality of the included literature was assessed using the quality evaluation criteria for cross-sectional studies recommended by the Agency for Healthcare Research and Quality (AHRQ) [24]. The scale comprises 11 criteria [25], yielding a total score ranging from 0 to 10 points. Responses marked affirmatively were scored as 1, whereas those marked as negative or unclear received a score of 0. Studies scoring 0–3, 4–7, and 8–11 points were classified as low, medium, and high quality, respectively. The Newcastle-Ottawa Scale (NOS) [26] was employed to evaluate the cohort study. The scale ranged from 1 to 9, with scores of 1 to 3 indicating low quality, 4 to 6 moderate quality, and 7 to 9 high quality. Studies with low-quality ratings were excluded from this analysis.

### Statistical methods

Herein, the analysis was conducted using Stata 17.0 software, involving indicators including the incidence of CKD-ap with its 95% CI, and the OR of associated factors with their corresponding 95% CI. To assess the heterogeneity among the included studies, both I² and Q tests were performed to quantify heterogeneity. *P* ≥ 0.1 and I² < 50% indicated minimal heterogeneity, warranting the application of a fixed-effect model. Conversely, *P* <0.1 and I² ≥ 50% indicated substantial heterogeneity, necessitating the adoption of a random-effects model. Subsequently, patients were stratified by dialysis type, assessment tool, region, and publication year. Subgroup and meta-regression analyses were performed to elucidate the heterogeneity sources. A sensitivity analysis was conducted to evaluate the robustness of the results. An Egger’s test was employed to assess publication bias among studies with at least ten entries. *P* > 0.05 indicated robust results and a low level of publication bias, whereas *P* < 0.05 suggested a significant difference.

## Results

### Literature selection steps and results

**Fig. 1:**
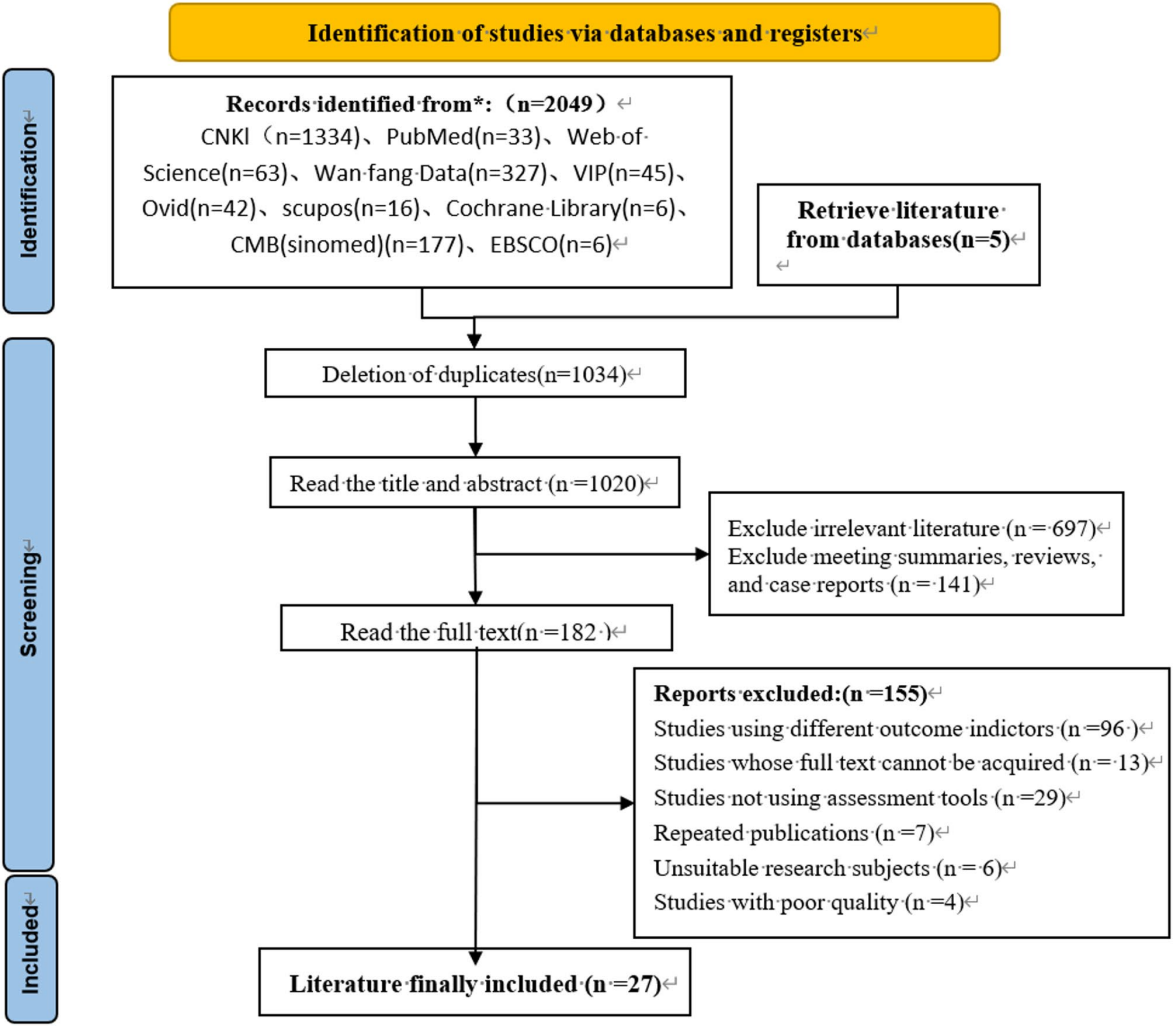
Literature selection process. *Consider, if feasible to do so, reporting the number of records identified from each database or register searched (rather than the total number across all databases/registers). **If automation tools were used, indicate how many records were excluded by a human and how many were excluded by automation tools.

As shown in Fig. 1, a total of 2,054 articles were retrieved from the database, ultimately leading to the inclusion of 27 [10, 22, 27–51] studies spanning 13 provinces, regions, municipalities, and special administrative regions. These studies involved a total of 10,346 patients, among whom 5,968 were diagnosed with CKD-ap (Fig. 1).

### Basic characteristics of the included studies and the results of the risk of bias evaluation

Table 2 presents the basic characteristics of the 27 included studies [10, 22, 27–51], comprising 1 cohort study [49] and 26 cross-sectional studies [12, 22, 27–48, 50, 51]. The qualitative assessment indicated a literature quality assessment (DQA) score of 8 for the cohort study and 7–10 for cross-sectional studies. Additionally, the analysis included 30 potential influencing factors (Table 2).

**Table 2:** Basic characteristics and evaluation results of included literature

Study	Study Type	Research location	Age (years)	Dialysis cycle (months)	Assessment tool	Sample size/number of itchers (*n*)	Prevalence (%)	Influencing factor	AHRQ
Liu 2017 [27]	I	Zhejiang	>18	>3	⑤	217/101	46.5	5、7、6、10	7
Sheng 2014 [28]	I	Shanghai			②	589/182	30.9	7、8、12	7
Chen 2022 [29]	I	Fujian	≥ 18	>3	①	120/56	46.7	6、8、10、11、12、13、27	9
Bao 2022 [12]	I	Shanghai	>18	≥ 3	②	1540/1192	77.4	6、9、10、19、20、21;23、24、25、	10
Wang 2007 [30]	I	Shanghai	23~82	≥ 3	②	190/93	49.0	1、22、	9
Shao 2021 [31]	I	Jiangsu	≥ 18	≥ 6	②	91/68	74.7	3、12	7
Bao 2021 [32]	I	Guangdong	≥ 18	≥ 3	⑤	121/76	62.8	11、18	7
Duan 2023 [33]	I	Sichuan	≥ 18	≥ 6	①+③	227/153	67.4	1、11、14、16	7
Hu 2019 [34]	I	Guangdong	39–68	>3	⑤	128/85	66.4	1、5、8、9、12、22	10
Luo 2020 [35]	I	Sichuan	40–85	≥ 6	②+③	316/110	34.8	1、9、10 、12、13、17	9
Li 2019 [36]	I	Jiangsu	≥ 18	>6	②	212/138	65.1	4	8
Jiang 2023 [37]	I	Jiangxi	≥ 18	≥ 3	④	100/63	63.0	5、8、10、12、22、27	7
Gong 2023 [38]	I	Jiangxi	—	—	②+③	80/80	100	4、5	8
Zhao 2024 [39]	I	Beijing	≥ 18		②+③	100/45	45	1、5、9、11、12、13、22、27、28	8
Liu 2022 [40]	I	Fujian	≥ 18	>3	②	202/77	38.1	——	7
Zhou 2021 [41]	I	Hubei	18–75	>3	①+⑥	214/214	100	10、27	9
Xia 2009 [42]	I	Guangdong	22–86		②	238/132	55.5	7、8、9、12、15、16、	8
Wei 2022 [22]	I	Sichuan	≥ 18	≥ 3	③	670/337	50.3	1、3、8、12、13	7
Wang 2024 [43]	I	Anhui	≥ 18	≥ 3	①+②	3025/1915	63.3	6、26	8
Chang 2022 [44]	I	Taiwan	>20		③	160/68	42.5	——	10
Zhao 2021 [45]	I	Zhejiang	20–90	≥ 1	①+③	148/60	40.5	11、12	8
HU 2019 [46]	I	Chongqing	—	>1	②	179/121	67.6	7、8、9、12、15、16	10
Xie 2021 [47]	I	Beijing	≥ 18	≥ 3	①+③	269/110	40.9	1、6、7、8	8
Hon-Yen 2016 [48]	I	Taiwan	≥ 20		①+②	380/137	36.1	1、8、14	8
Ko 2013 [49]	II	Taiwan	—	≥ 3	②	321/111	34.6	——	8
Ma 2019 [50]	I	Henan	—	>3	④	352/161	45.7	10、11、12、13、17、30	8
Wang 2024 [51]	I	Henan	≥ 18	≥ 3	①	198/108	54.6	1, 6, 10, 27	9

Note: Study Type: I: cross-sectional study; II: cohort study. Assessment tool: ① Diagnostic criteria for uremic pruritus [3];② VAS;③ 5D~IS;④ Duo’s itch score;⑤Dirk R Kuypers scale;⑥ 12~PSS. Associated factors: 1 Age; 2 gender; 3 Smoking history; 4 Sleep quality; 5 Dialysis duration; 6 Dialysis type; 7 Blood calcium (Ca); 8 Blood phosphorus (P); 9 Ca-P product; 10 Urea clearance index (Kt/v); 11 C-reactive protein (CRP); 12 Intact parathyroid hormone (iPTH); 13 β2-microglobulin (β2-MG); 14 Triglycerides (Tr); 15 Blood urea nitrogen (BUN); 16 Serum creatinine (SCr); 17 Primary diseases;18 Whole blood manganese (Mn); 19 Anemia; 20 Heart failure; 21 CKD-MBD; 22 Xerosis; 23 P-containing binder; 24 calcimimetics; 25 Calcitriol; 26 Anti-itch drug for external use; 27 TNF-ɑ;28 IL-6;29 Uric acid; 30 Leptin

### Meta-analysis of the incidence of CKD-ap

#### Incidence of CKD-ap

A meta-analysis was conducted to assess the incidence of CKD-ap as reported by the 27 included studies. Significant heterogeneity was observed among the studies (*I²*= 97.5%, *P* < 0.001). Therefore, a random-effects model was employed. The meta-analysis results demonstrated a total incidence of CKD-ap of 52% in China (95% CI = 46.0%~59.0%). In addition, a meta-analysis was further conducted to assess the prevalence of different degrees of itching [12, 22, 27, 30, 32, 33, 38–44, 47, 48]. The results indicated that the prevalence of mild itching was 26% (I²=98.9%, 95% CI: 16-36%, *P* < 0.001), moderate itching was 22% (I²=98.2%, 95% CI: 16-28%, *P* < 0.001), and severe itching was 8% (I²=92.8%, 95% CI: 6-10%, *P* < 0.001) (Figs shown in S1-A).

#### Subgroup analysis

The patients were grouped by the publication year, region, and itch scale (Table 3).

1. Research region: The incidence of CKD-ap in North China, East China, South China, Central China, and Southwest China was 42% (95% CI = 36.9,47), 53.3% (95% CI = 42.6,64.0), 52.4%(95% CI = 40.4,64.5), 49.8% (95% CI = 45.2,54.3), and 54.9% (95% CI = 40.7,69.2), respectively.
2. Publication year: The incidence of CKD-ap before 2010, from 2010 to 2019, and from 2020 to 2024 was 52.6% (95% CI = 47.9,57.3), 48.9%(95% CI = 38.8,59.0), and 53.5%(95% CI = 46.0,61.0), respectively.
3. Assessment tool: The incidence of CKD-ap assessed using the diagnostic criteria for CKD-ap, Visual Analog Scale (VAS), Duo’s itch score, Dirk R. Kuypers scale, and 12-Item Psoriasis Symptom Scale (12-PSS) was 54.7% (95% CI = 46.4,62.9), 51.6% (95% CI = 41.8,61.6), 46.0% (95% CI = 37.9,54.1), 50% (95% CI = 45.2,54.3), 58.4% (95% CI = 45.6,71.2), respectively.
4. Study type: The prevalence was 53.0% (95% CI = 47.0,59.0) in cross-sectional studies and 34.6% (95% CI = 29.4,40.1) in longitudinal studies.

**Table 3:** Subgroup analysis of the prevalence of CKD-ap in MHD patients in China

Item	Number of studies included	*I*^2^ value	Model	Incidence (95%CI) (%)	*P*
**Region**					
North China	2	0	Fixed	42(36.9,47)	<0.001
East China	11	98.5	Random	53.3(42.6,64.0)	<0.001
South China	6	93.5	Random	52.4(40.4,64.5)	<0.001
Central China	4	0	Fixed	49.8(45.2,54.3)	<0.001
Southwest China	4	96.5	Random	54.9(40.7,69.2)	<0.001
**Publication year**					
2000~2009	2	0	Fixed	52.6(47.9,57.3)	<0.001
2010~2019	8	96.3	Random	48.9(38.8,59.0)	<0.001
2020~2024	17	97.6	Random	53.5(46.0, 61.0)	<0.001
**Assessment tool**					
Diagnostic criteria of UP	7	93.9	Random	54.7(46.4,62.9)	<0.001
VAS	14	98.6	Random	51.6(41.8,61.6)	<0.001
5D~IS	8	92	Random	46.0(37.9,54.1)	<0.001
Duo’s itch scale	2	0	Random	50(45.2,54.3)	<0.001
Dirk R Kuypers scale	3	0	Fixed	58.4(45.6,71.2)	<0.001
12~PSS	1	0	-	—	<0.001
**Study Type**					
cross-sectional study	26	97.4	Random	53(46,59)	<0.001
Longitudinal study	1	-	-	34.6(29.4,40.1)	<0.001

#### Sensitivity analysis and meta-regression analysis

To evaluate the robustness of the association results, sensitivity analyses were conducted by sequentially excluding individual studies and recalculating the pooled prevalence. The analyses included the total prevalence, and prevalence of different severities of CKD-aP, and subgroup analyses with high heterogeneity (focusing on studies with more than two observations) following the methods described above. The results demonstrated that the combined p-value remained stable, confirming the robustness and reliability of the findings. Additionally, meta-regression analyses of CKD-ap prevalence were conducted across more than 10 studies, with year of publication, region, assessment tools, and study type as covariates. The prevalence showed no significant changes, further supporting the stability and reliability of the findings (Table 4).

**Table 4:** Meta-regression analysis of the prevalence of CKD-ap in MHD patients in China

	*b(95%CI)*	*SE*	*t* value	*P* value
**Total prevalence**				
Region	0.023(-0.067,0.113)	0.434	0.53	0.602
Publication year	-0.015(-0.032,0.061)	0.023	-0.66	0.519
Assessment tool	0.002(-0.041,0.044)	0.021	-0.07	0.942
Study Type	-0.090(-0.230,0.050)	0068	-1.33	0.196
**Eastern China region**				
Publication year	0.045(-0.155,0.244)	0.084	0.53	0.613
Assessment tool	-0.012(-0.163,0.139)	0.068	-0.18	0.861
**2020–2024**				
Region	0.004(-0.058,0.661)	0.285	0.14	0.891
Assessment tool	-0.003(-0.057,0.063)	0.028	0.09	0.927
**VAS**				
Region	-0.016(-0.126,0.093)	0.048	-0.33	0.746
Publication year	0.032(-0.138,0.203)	0.075	0.43	0.678
Study Type	-0.185(-0.561,0.190)	0.171	-1.09	0.301
**Cross-sectional study**				
Region	0.014(-0.035,0.063)	0.023	0.60	0.552
Publication year	0.023(-0.086,0.131)	0.052	0.44	0.665
Assessment tool	0.006(-0.035,0.047)	0.020	0.31	0.756

#### Risk of publication bias in CKD-ap prevalence

Egger’s test was conducted on subgroup analyses with high heterogeneity to assess publication bias among the included studies. Notably, the p-values for the prevalence of the outcomes were generally greater than 0.05, except for studies published from 2010 to 2019 (*P* = 0.004), 2020 to 2024 (*P* = 0.031), those conducted in South China (*P* = 0.036), and cross-sectional studies (*P* = 0.035). These findings indicated potential publication bias in studies published between 2010 and 2019, those conducted in South China, and cross-sectional studies from 2020 to 2024 (Figs shown in S1-B).

### Meta-analysis of CKD-ap-associated factors in Chinese MHD patients

#### Meta-analysis of CKD-ap-associated factors

The CKD-ap-associated factors discussed in the 27 studies included were analyzed. Studies that examined 2 or more factors were included in the meta-analysis. Dialysis modality, KT/V, and serum calcium were identified as factors associated with a reduction in CKD-aP, whereas duration of dialysis, diabetes mellitus, dry skin, serum phosphorus, calcium-phosphorus product, C-reactive protein, iPTH, β2-MG, and serum creatinine (SCr) were identified as factors associated with an increased risk of CKD-aP in maintenance hemodialysis patients (Table 5).

**Table 5:** Meta-analysis of factors influencing CKD-ap in MHD patients in China

	Number of studies included	Sample size	*I* ^2^	Model	*OR (95%CI)*	*P*	Sensitivity
Sociodemographic data							
Age	9	2317	86.0	Random	0.99(0.65,1.33)	<0.001	0.99(0.65,1.33)
Length of dialysis treatment	5	625	88.4	Random	1.51 (1.15, 1.88)	<0.001	1.51 (1.15, 1.88)
Dialysis type	5	5152	24.4	Fixed	0.54(0.23, 0.85)	<0.001	0.54(0.23, 0.85)
Primary disease	5	2404	80.7	Random	1.43 (0.87, 1.99)	<0.001	1.43 (0.87, 1.99)
Xerosis	4	518	0	Fixed	2.46 (1.74, 3.19)	<0.001	2.46 (1.74, 3.19)
Biochemical indicators							
Blood Ca	3	1007	0	Fixed	0.33(0.31,0.35)	<0.001	0.33(0.31,0.35)
Blood P	7	1735	91.3	Random	1.18 (0.55, 1.81)	<0.001	1.18 (0.55, 1.81)
Ca-P product	5	2263	75.8	Random	2.18 (1.14, 3.22)	<0.001	2.18 (1.14, 3.22)
KT/V	8	3057	85.8	Random	0.60 (0.24,0.96)	0.009	0.60 (0.24,0.96)
CRP	6	1068	84.9	Random	1.14(0.78, 1.51)	<0.001	1.14(0.78, 1.51)
iPTH	11	2674	98.8	Random	2.45 (0.81,4.09)	<0.001	2.45 (0.81,4.09)
β_2_-MG	4	888	70.6	Random	2.24 (0.94, 3.53)	<0.001	2.24 (0.94, 3.53)
SCr	2	406	0	Fixed	1(1.001,1.005)	<0.001	1.003(1.001, 1.005)

#### Meta-regression analysis

Meta-regression was performed on over 10 publications for the influencing factors. Region, year of study, assessment tool, and study type were hereby utilized as covariates to explore the heterogeneity of parathyroid hormone (iPTH), and the results demonstrated study type as a significant source of iPTH heterogeneity (Table 6).

**Table 6:** Meta-regression analysis of CKD-ap-associated factors

	*b(95%CI)*	*SE*	*t* value	*P* value
**iPTH**				
Region	0.084(-0.497,0.665)	0.246	0.34	0.742
Publication year	0.215(-0.425,0.854)	0.270	0.79	0.454
Assessment tool	-0.091(-0.889,0.707)	0.370	-0.25	0.809

#### Sensitivity analysis

(1) A random-effects or fixed-effects model was employed for the sensitivity analysis of the associated factors. The results indicated no significant changes in the study outcomes, suggesting robust findings.

(2) Sensitivity analyses were performed to assess the robustness of the meta-analysis findings on influencing factors by sequentially excluding studies with I² > 50% and those involving more than two articles related to influencing factors. For example, the “HU et al.” [34] study remarkably contributed to the heterogeneity associated with the calcium-phosphorus product. Exclusion of this study resulted in a combined OR of 2.56 (1.99, 3.13), with a significant reduction in heterogeneity (I²=14.8%, p = 0.318). Similarly, the “Ma et al. [50]” study was a major contributor to β2 microglobulin heterogeneity, and its exclusion led to a combined OR of 2.84 (1.81, 3.87), accompanied by a complete elimination of heterogeneity (I²=0%, p = 0.468). Exclusion of these factors revealed no significant variation in the combined OR of other influencing factors, thereby confirming the robustness and reliability of the findings.

#### Publication bias analysis of influencing factors

Egger’s test was carried out to assess publication bias for studies with over articles, with a P value < 0.05 indicating the presence of publication bias. In this study, Egger’s test was applied to the influencing factors exhibiting high heterogeneity, revealing that the P-values for KT/V (*P* = 0.027) and duration of dialysis treatment (*P* = 0.006) were less than 0.05. This indicated the presence of publication bias affecting the urea clearance coefficient and the duration of dialysis treatment. Apart from these findings, no evidence of publication bias was detected for the other influencing factors (Figs shown in S1-C).

## Discussion

### Incidence of CKD-ap in MHD patients

The meta-analysis indicated that the prevalence of itchy skin among Chinese hemodialysis patients was 52%, aligning with findings from an international survey on the prevalence of itchy skin in hemodialysis patients [9]. However, this prevalence was considered high when compared to findings from domestic survey studies on the prevalence of itchy skin in hemodialysis patients [8]. The prevalence of mild, moderate, and severe CKD-ap in Chinese hemodialysis patients was 26%, 22%, and 8%, respectively. These findings were comparable to those of the European Quality (EQUAL) study [52], despite the higher prevalence of moderate and severe CKD-ap than that reported in the study by Wang et al. [43]. The subgroup analysis results in this study revealed significant differences associated with variations in the year of study, region, assessment tool, and study design. It was observed that the prevalence of pruritus was highest in Southwest China (54.9%), with the lowest prevalence reported in North China (42%). These regional differences might be attributed to factors such as climate, dietary habits, and lifestyle. In terms of the year of study, the prevalence of pruritus in dialysis patients increased significantly from 53.5% in the period from 2020 to the present, compared to 52.6% before 2010 and 48.9% from 2010 to 2019. This increase might be attributed to advancements in medical technology having extended the life expectancy of patients with end-stage renal disease. Over time, the focus of healthcare has shifted from merely managing the patients’ diseases to also improving their quality of life and addressing both their physical and mental health. Additionally, the necessity for timely prevention and intervention of complications from long-term dialysis has become increasingly recognized. However, this rise in prevalence may also be influenced by the quality of the included literature and the sample size. Regarding assessment tools: Assessment using the Dirk R. Kuypers Pruritus Assessment Scale revealed the highest prevalence of pruritus (58.4%), whereas the 5D-IS scale demonstrated the lowest prevalence (50%). The significant heterogeneity observed between these measures (*I²=*91.2%, *P* < 0.001) was primarily attributed to substantial methodological differences in the assessment tools. To resolve these inconsistencies, standardized diagnostic protocols should be implemented, and rigorously designed multicenter trials should be conducted. Notably, there is currently no uniform standard for assessing itch in dialysis patients, both domestically and internationally, with the CKD-ap diagnostic criteria [47] being the most commonly used. However, there is no effective assessment tool to evaluate the site, frequency, and severity of itch in these patients. Some studies have indicated that the visual analog scale (VAS) is the most extensively adopted clinical tool for assessing unidimensional itchy skin. Additionally, this scale is often employed in conjunction with other multidimensional scales or diagnostic criteria for itchy skin in various studies [48]. Herein, the prevalence observed in the cohort study (34.6%) was significantly lower than that reported in the cross-sectional study (53%). One possible explanation could be the loss-to-follow-up and higher mortality rates observed among patients in cohort studies. For instance, Grochulska et al. [53] reported a one-year mortality rate of approximately 15.3% in their study on pruritus-related mortality among hemodialysis patients. Furthermore, in a Chinese study by Ko et al. [49] investigating CKD-aP among hemodialysis patients, only 35% of the participants completed the 5-year follow-up. Conversely, the limited number of cohort studies identified in the existing literature could introduce selection bias and undermine the reliability of prevalence estimates for CKD-ap in Chinese maintenance hemodialysis patients.

### Associated factors

#### Sociodemographic factors

The present findings demonstrated that age, dialysis duration, and modality significantly impacted the prevalence of CKD-ap. Advanced age was observed to be an independent risk factor for CKD-ap in patients undergoing maintenance hemodialysis, possibly attributed to the progressive decline in skin barrier function with aging [54], which led to increased trans-epidermal water loss and accelerated degeneration of the stratum corneum. As a result, the skin became increasingly dry, thereby worsening the severity of pruritus [55]. Moreover, the duration of dialysis treatment was found to be a significant risk factor for CKD-ap in patients undergoing maintenance hemodialysis. Longer dialysis duration was associated with a higher prevalence of CKD-ap. Chronic dialysis led to the accumulation of macromolecular toxins in the body, resulting in the elevation of extracellular free divalent ions in the basal epidermal layer in long-term hemodialysis patients. Furthermore, the retention of calcium phosphate salts in the skin and other tissues activated local nerve fibers, thereby contributing to the development of pruritus [56]. In contrast, the dialysis modality acted as a protective factor against Chronic Kidney Disease-associated Pruritus (CKD-aP) in patients undergoing maintenance hemodialysis. Uremic toxins were categorized into three groups based on molecular weight, namely small, medium, and large molecules. The study by Bao et al. [12] showed comparable prevalence of pruritus in patients undergoing hemodialysis (HD) alone and those receiving a combination of hemodialysis and hemoperfusion (HD + HP). However, the prevalence of moderate to severe Chronic Kidney Disease-associated Pruritus (CKD-aP) was lower in the HD + HP group compared to the HD group. Li et al. [57] conducted a clinical study on Chronic Kidney Disease-associated Pruritus (CKD-aP) and demonstrated that hemoperfusion effectively removed inflammatory mediators as well as large and medium molecular weight toxins from patients with septic acute kidney injury. However, HD effectively removed water-soluble and low protein-binding substances and corrected water-electrolyte imbalances, yet it was less efficient in clearing medium- and large-molecule toxins, resulting in inadequate clearance of toxins associated with end-stage renal disease. In contrast, hemoperfusion (HP) does not correct water-electrolyte or acid-base imbalances, yet it is highly effective in removing medium- and large-molecule toxins, as well as protein-bound toxins. Consequently, in most cases, patients are recommended to undergo a combination of HP and HD to eliminate toxins from the body. This approach effectively removes small-molecule toxins through hemodialysis and medium- and large-molecule toxins via hemoperfusion, thereby enhancing toxin clearance, increasing metabolic rate, and improving both inflammatory responses and pruritic symptoms in patients [58–60]. In daily life, healthcare professionals should recommend that patients and their families monitor environmental temperature and humidity, use emollients or coolants to relieve dry skin, and choose soft clothing to preserve skin integrity. In the hospital setting, acupuncture and compression therapies, including electroacupuncture, warm needling, and auricular pressure bean therapy, may be used in addition to pharmacological interventions. Among these, auricular pressure bean therapy, boasting its low cost, non-invasive nature, high patient acceptance, and ease of administration, has proven effective in reducing itching. It is recommended that in future clinical practice, healthcare professionals, including physicians and nurses, strengthen risk assessment of CKD-ap and improve health education and intervention strategies for these patients. The goal is to ensure early prevention, detection, and intervention, thus minimizing the risk of exacerbations.

#### Physiological indicators

The findings of this study indicated calcium, phosphorus, KT/V, C-reactive protein, iPTH, β2-MG, BUN, and SCr as independent risk factors for CKD-ap. Serum calcium was hereby identified as a protective factor for CKD-ap, whereas phosphorus was recognized as a risk factor. However, several other studies have suggested that both serum calcium and phosphorus may act as risk factors for CKD-ap [56, 61–62]. Uremic patients generally exhibit calcium and phosphorus metabolic disorders, primarily characterized by hypocalcemia and hyperphosphatemia. These disturbances lead to elevated parathyroid hormone (PTH) levels, triggering SHPT and further increasing PTH secretion. This cascade results in systemic inflammation and skin dryness and may even directly stimulate histamine release from mast cells via neural pathways, thereby causing pruritus [63]. Kt/V is a key metric for evaluating dialysis adequacy, with values ≥ 1.5 considered optimal for effective dialysis [64]. The study findings demonstrated Kt/V as a protective factor against CKD-ap. As Kt/V increases, toxin levels in the patient’s body decreased, thereby alleviating CKD-ap [65]. Notably, SCr, the most commonly used marker for assessing glomerular function and dialysis adequacy, was considered a protective factor against CKD-ap. Inadequate dialysis led to a significant increase in SCr levels, indicating insufficient dialysis and leading to toxin accumulation in the body [66]. Timely adjustment of the dialysis regimen could indirectly improve CKD-ap in patients. In addition, CRP, iPTH, β2-MG, and BUN were considered risk factors for CKD-ap. CRP acted as an inflammatory marker reflecting changes in glomerular filtration rate (GFR). A decrease in CRP levels was associated with reduced urea clearance, leading to toxin accumulation and subsequent skin itching [66]. β2-MG served as a well-established marker of renal damage [67], and BUN was considered a primary indicator for assessing renal function [66]. Elevated levels of iPTH, β2-MG, and BUN not only activated inflammatory pathways and increased the release of inflammatory factors such as IL-6 and TNF-α but also stimulated sensory nerve endings, thereby triggering pruritus. Additionally, these factors impaired skin barrier function, leading to increased transepidermal water loss, skin dryness, and worsening of pruritus [66–68]. These biochemical indicators suggested that insufficient dialysis led to increased toxin accumulation in the body, exacerbating CKD-ap. Therefore, during clinical treatment, healthcare providers should administer antihistamines such as loratadine and gabapentin, and advise patients on daily diet, skin care, and appropriate exercise. These measures enhance metabolism and reduce mast cell activation, alleviating skin itching. Additionally, patients should be encouraged to attend regular outpatient follow-ups and tests to monitor biochemical indices, ensuring that they remain within acceptable ranges. Complementary traditional Chinese medicine therapies, such as herbal baths, acupuncture, and compression therapy, may also facilitate to alleviate pruritic symptoms.

### Limitations and shortages

This study aimed to examine the prevalence of CKD-ap and its associated factors among Chinese patients undergoing MHD. With a large, multi-regional sample size, this study offers a valuable reference for future research on CKD-ap and relevant interventions. However, this study is still subjected to several limitations. First, the cross-sectional design may be susceptible to confounding factors. Second, the use of diverse assessment tools across studies could have influenced the final results. Furthermore, the meta-analysis revealed negative final outcome odds ratios (ORs) for smoking history, sleep quality, triglycerides (TG), and blood urea nitrogen (BUN). Moreover, age may not be considered statistically significant, given its proximity to 1 and the fact that the confidence intervals include 1 in this meta-analysis.Despite repeated statistical analyses and logarithmic transformations, these effects could not be eliminated and were subsequently excluded. This may be attributed to the significant variability in data distribution and the relatively small sample size. Moreover, factors mentioned in fewer than two studies were excluded from the aggregate analysis. Future longitudinal studies, those with larger sample sizes and choose more authoritative and specialized diagnostic criteria for Chronic Kidney Disease-associated Pruritus (CKD-aP) should be conducted to yield more reliable data and validate the findings of this study.

## Conclusion

Among Chinese patients undergoing MHD, the prevalence of CKD-ap was 52%, with 26% experiencing mild, 22% moderate, and 8% severe CKD-ap. The prevalence showed an increasing trend. The primary risk factors included age, duration of dialysis, primary disease, dry skin, serum calcium (Ca), serum phosphorus (P), calcium-phosphorus product, C-reactive protein, intact parathyroid hormone (iPTH), β2-microglobulin (β2-MG), blood urea nitrogen (BUN), and serum creatinine (SCr). Additionally, dialysis modality and KT/V were identified as protective factors against pruritus in Chinese hemodialysis patients. Consequently, clinicians should closely monitor patients with CKD-ap and promptly identify high-risk individuals based on the aforementioned factors. Medical professionals should encourage regular check-ups and hospital visits for these patients, while also developing and implementing timely, individualized interventions and treatment strategies to reduce the incidence and slow the progression of CKD-ap. Additionally, patients should be encouraged to adopt healthier lifestyles, and the development of specialized medications with minimal side effects for MHD patients with CKD-ap should be prioritized to improve their quality of life.

## Electronic supplementary material

Below is the link to the electronic supplementary material.

Supplementary Material 1

## Data Availability

No datasets were generated or analysed during the current study.
