# Supplementary material for: Prevalence and influencing factors of pruritus in maintenance hemodialysis patients in China: a meta-analysis
Source: BMC Nephrol. 2025 May 29;26:266. doi: 10.1186/s12882-025-04163-7 (PMC12123892; doi:10.1186/s12882-025-04163-7)
Supplement: Supplementary file 1 — Supplementary Material 1 [file 12882_2025_4163_MOESM1_ESM.docx]

**A:Forest plot of prevalence**

| **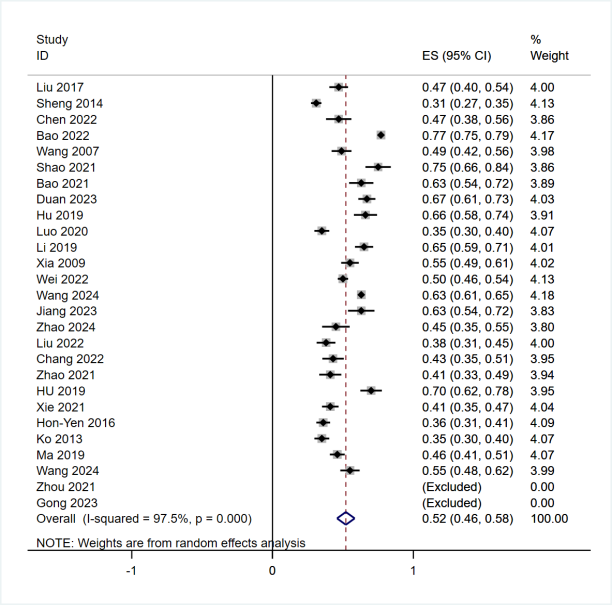** |  |
| --- | --- |
| **(a)total prevalence** | **(b)Prevalence of mild uremic pruritus** |
| **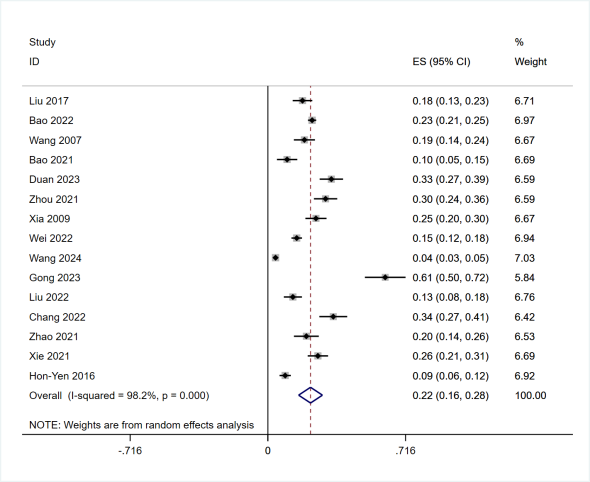** | **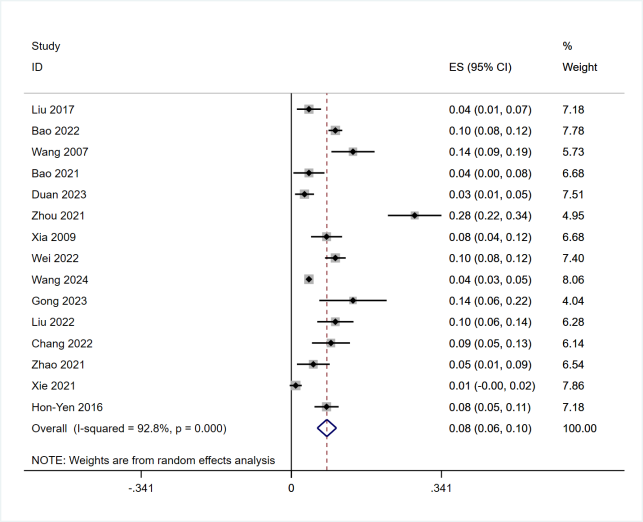** |
| **(c)Prevalence of moderate uremic pruritus** | **(d)Prevalence of severe uremic pruritus** |

## **B：Funnel plot of Uremic pruritus prevalence**

|  |  |
| --- | --- |
| **(a)2010-2019** | **(b)2020-2024** |
|  |  |
| **(c)Southern China** | **(d)Cross-sectional studies** |

**C:Funnel plot of influencing factors**

|  |  |
| --- | --- |
| **(a)KT/V** | **(b)Length of dialysis treatment** |
